# Starch Hydrolysis, Polyphenol Contents, and *In Vitro* Alpha Amylase Inhibitory Properties of Some Nigerian Foods As Affected by Cooking

**DOI:** 10.3389/fnut.2017.00060

**Published:** 2017-12-04

**Authors:** Sani Saidu, Chinedum Ogbonnaya Eleazu, David Ebuka, Anthony Ikechukwu, Montalto Blessing, Nwoye Chibuike, Chukwu Chukwuma

**Affiliations:** ^1^Federal University Ndufu-Alike, Ikwo, Nigeria

**Keywords:** type 2 diabetes, starch digestibility, starch blockers, functional foods, potato, alpha-amylase

## Abstract

The effect of cooking on starch hydrolysis, polyphenol contents, and *in vitro* α-amylase inhibitory properties of mushrooms (two varieties *Russula virescens* and *Auricularia auricula-judae*), sweet potato (*Ipomea batatas*), and potato (*Solanum tuberosum*) was investigated. The total, resistant, and digestible starch contents of the raw and cooked food samples (FS) ranged from 6.4 to 64.9; 0 to 10.1; and 6.4 to 62.7 g/100 g, respectively, while their percentages of starch digestibility (DS values expressed as percentages of total starch hydrolyzed) ranged from 45.99 to 100. Raw and boiled unpeeled potato, raw and boiled peeled potato, raw *A. auricula-judae*, and sweet potato showed mild to high α-amylase inhibition (over a range of concentration of 10–50 mg/mL), which was lower than that of acarbose (that had 69% inhibition of α-amylase over a range of concentration of 2–10 mg/mL), unlike raw *R. virescens*, boiled *A. auricula-judae*, and boiled sweet potatoes that activated α-amylase and boiled *R. virescens* that gave 0% inhibition. The FS contained flavonoids and phenols in addition. The significant negative correlation (*r* = −0.55; *P* = 0.05) between the α-amylase inhibitory properties of the raw and cooked FS versus their SD indicates that the α-amylase inhibitors in these FS also influenced the digestibility of their starches. In addition, the significant positive correlation between the α-amylase inhibitory properties of the raw and cooked FS versus their resistant starch (RS) (*r* = 0.59; *P* = 0.01) contents indicates that the RS constituents of these FS contributed to their α-amylase inhibitory properties. The study showed the usefulness of boiled unpeeled potato, boiled potato peeled, and raw sweet potato as functional foods for people with type 2 diabetes.

## Introduction

One of the therapeutic approaches that have been found to be useful in the management of type 2 diabetes is reduction of postprandial hyperglycemia through inhibition of the carbohydrate hydrolyzing enzymes, such as α-amylase in the digestive tract (1). Synthetic drugs such as acarbose and others that are currently being used to inhibit these enzymes, tend to be non-specific in targeting different glycosidases, thereby producing adverse effects. This has, therefore, led to increased search for safer, specific, and effective hypoglycemic agents from either dietary or herbal sources (2).

Many functional foods have been used successfully by mankind since ancient era to counteract diabetes and its associated complications. These foods are widely prescribed even when their biologically active compounds are unknown, because of their safety, effectiveness, and availability (3).

Starch digestibility (SD) is the proportion of starch that is digested under certain conditions. Several factors influence the digestibility of starches in foods such as: carbohydrate contents of foods, nutritional composition of starch [rapidly digestible starch, slowly digestible starch, and resistant starch (RS)], method of cooking foods, and others. These factors tend to affect the change in blood glucose after eating a meal (4).

Mushrooms refer to macro fungi with distinctive fruiting bodies that are large enough to be seen with the naked eyes (5). Numerous species of mushrooms exist in nature and among the 22,000 known species, only a few are edible. Mushrooms not only serve as useful sources of nutrients but they have also been reported to be therapeutic foods that are useful in preventing several diseases such as diabetes, cancer, etc. These functional characteristics of mushrooms were associated with the presence of high molecular weight compounds such as polysaccharides, proteins, and lipids as well as a number of low molecular weight metabolites in them such as phenols, lectins, alkaloids, lactones, etc. (5).

*Russula virescens* is an edible mushroom that is found in different countries of the world. It is considered to be medicinal and one of the best of the genus-*Russula* (6).

*Auricularia auricula-judae* is a species of edible Auriculariales fungus that is found worldwide and is also considered to be medicinal (7).

While studies have reported the blood glucose lowering potentials of *A. auricula-judae* (7) and extracts of the fruit bodies of *R. virescens* (8), there is paucity of information in literature on the effect of boiling of these mushrooms (which is the form in which they are eaten in typical diets) on blood glucose response and the activities of starch blockers that could naturally be present in them.

Sweet potato (*Ipomea batatas* L) is an important and highly nutritious food crop that is grown in most parts of the world, especially in the tropics where the bulk of the crop is cultivated and consumed (9). Many studies have reported different medicinal potentials of sweet potato. These properties have been attributed to either a single or combined effect of the phytochemicals that are present in the plant (10). However, with regards to its relevance in the management of type 2 diabetes, contradictory reports were given in literature. For instance, while Dutta (11) and Zakir et al. (12) reported the beneficial role of sweet potato in glycemic control in type 2 diabetic subjects, Jonathan et al. (13) reported sweet potato to be an intermediate glycemic index (GI) crop while the study by Kaniz et al. (14) on normal and diabetic subjects suggested it to be a high GI crop. In addition, there is paucity of information in literature on the effect of boiling (which is one of the forms in which sweet potato is eaten in typical African diets) on the digestibility of the starches in sweet potato. This is especially important as the method of cooking of foods is one of the factors that affect the digestibility of starches in foods, which could also affect the blood glucose responses to these foods when eaten (15). Furthermore, there is paucity of information in literature on the effect of cooking of sweet potato on the activities of starch blockers that could be present in it.

Potato (*Solanum tuberosum*) serves as the major source of energy in many societies. It is a carbohydrate-rich food that is prepared and served in a variety of ways. In typical African diets, potatoes are eaten either with or without the peels. The predominant form of the carbohydrate in potato tubers is starch (16) but a significant portion of this starch is resistant to digestion by enzymes in the stomach and small intestine.

Despite the presence of considerable levels of RS in this plant, information in literature regarding the role of potatoes in the management of diabetes mellitus gave contradictory reports. For instance, studies conducted on the glycemic indices of potatoes in different countries varied widely. Whereas a high GI was reported for potatoes in Australia, Canada, and New Zealand, a medium GI was reported for potatoes in Romania, while a low to high GI was reported for potatoes in Kenya and India (14). Furthermore, there is scarcity of information in literature on the effect of cooking on the digestibility of the starches in sweet potato. This is especially true as potatoes are eaten in typical African diets either boiled or fried, with or without the peels and the method of cooking foods as earlier stated, is one of the factors that influence the digestibility of starches in foods, which in essence could affect the glycemic response to these foods when eaten. In addition, information on the effect of cooking of potato on the activities of starch blockers that could naturally be present in it is also scarce in literature. Moreover, studies (17) have shown a positive association between the polyphenols in some plant-based foods with their inhibitory actions on these starch digestive enzymes and these polyphenols being polar and heat labile, could also be affected by different food cooking techniques, which in essence could affect the inhibitory actions of the plant based foods on these starch digestive enzymes.

In line with these, the present study sought to investigate the effect of cooking on the digestibility of the starches in mushrooms (*R. virescens* and *A. auricula-judae*), sweet potato (*I. batatas*), and potato (*S. tuberosum*) and to determine the effect of cooking on the polyphenol (total phenols and flavonoids) contents of these food samples (FS) and their inhibitory actions on α-amylase *in vitro*.

## Materials and Methods

### Chemicals

Glucose assay kit (Sigma Catalog No: GAG020), pepsin (Sigma Catalog No: P6887), α-amylase from porcine pancreas (Sigma Catalog No: A3176), amyloglucosidase from *Aspergillus niger* (Sigma Catalog No: 10115), starch (Sigma Catalog No: S2004), 3.5-dinitrosalycyclic acid (Sigma Catalog No: D0550), maleic acid (Sigma Catalog No: M0375), hydroxymethyl aminomethane (Sigma Catalog No: 252859), gallic acid (Sigma Catalog: G7384), Quercetin (Sigma Catalog: 1592409), and Folin–Ciocalteu reagent (Sigma Catalog: F9252) that were used for this study were products of Sigma-Aldrich, Inc., USA. Other chemicals not included in the list above and which was used for this study was bought from Nigeria and was also of analytical grade.

### Plant Materials

The mushroom varieties (2 kg each) were obtained from Ikwo, Ebonyi State, Nigeria and they were identified as *R. virescens* and *A. auricula*-judae, respectively. The sweet potato (white fleshed) and potato (Variety-British Queens) samples (5 kg each) were bought from the meat market in Abakaliki town, Ebonyi State, Nigeria. The samples were thoroughly washed with distilled water. The sweet potatoes and some samples from the potatoes were peeled. All the samples from each plant were cut to small sizes.

### Sample Preparation

About 0.4 g of the raw FS were weighed into different tubes. Thereafter, the samples were cooked at 100°C for about 50–55 min for *R. virescens*, 45 min for *A. auricula*-judae, 30 min for sweet potato, 55 min for unpeeled potato, and 50 min for peeled potatoes, respectively, in 40 mL of distilled water, using a thermostatically regulated water bath. For the unprocessed (without boiling or peeling or both as applicable), samples that were and kept as the raw (Control), 0.4 g were prepared in 40 mL of distilled water. After the samples had been cooked, they were made up to 40 mL with distilled water, cooled under room temperature, and then homogenized in the liquids (water) they were dissolved in. The raw samples were homogenized in a similar way as the boiled samples. Analysis was carried out in the tubes in which the FS were homogenized. All analysis was carried out on fresh weight basis.

### Assay for Total Starch (TS)

The TS contents of the raw and cooked FS were assayed for, using the method of Goni et al. (18). Following preparation of the samples as described above, the homogenized samples were dispersed in 6 mL of 2 M KOH at room temperature for 30 min to solubilize all the starch. Thereafter, 60 µL of amyloglucosidase from *A. niger* (ref. 10115, Sigma, Germany) (in sodium acetate buffer, pH 4.6) was added and the reaction mixture was incubated for 45 min at 60°C in a water bath. After the incubation period, the FS were centrifuged for 10 min at 3,000 × *g*. Thereafter, the supernatants were collected and the concentration of glucose in each supernatant was determined using a glucose assay kit (ref. GAGO20, Sigma, Germany). The amount of glucose released was measured at 540 nm using a UV spectrophotometer against the reagent blank. The concentration of glucose (milligrams per milliliter) released was converted to starch by multiplying by 0.9 and the values were reported as g/100 g of sample.

### Assay for RS

The RS contents of the raw and cooked FS were also assayed for using the method of Goni et al. (18). After preparing the FS using the procedure as shown above, 0.2 mL of pepsin solution (from porcine gastric mucosa, 1 g in 10 mL HCl–KCl buffer, pH 1.5) was added and the mixtures were kept at 40°C for 1 h with constant shaking for protein removal. Thereafter, they were cooled at room temperature. Subsequently, 9 mL of 0.1 M Tris-maleate buffer, pH 6.9 was added, followed by 1 mL of a solution of α-amylase [from porcine pancreas (ref. A-3176, Sigma, Germany)] to enable starch hydrolysis. They were well mixed and incubated at 37°C for 16 h in a water bath. Following the incubation period, the samples were centrifuged at 3,000 × *g* for 15 min and the supernatants were discarded. The residues were collected and 3 mL of distilled water was added, followed by 6 mL of 2 M KOH to solubilize the starch and 60 µL of amyloglucosidase (in sodium acetate buffer, pH 4.6). The reaction mixture was kept in a water bath for 45 min at 60°C after which they were centrifuged for 10 min at 3,000 × *g*. The supernatants were collected and the amounts of glucose released were determined using the glucose assay kit, following the procedure that was reported for TS. The concentration of glucose released was converted to starch by multiplying by 0.9 and the values were reported as gram/100 g of sample.

Digestible starch (DS) was calculated as the difference between TS and RS contents respectively (19).

### Starch Digestibility

The SD of the raw and cooked FS was evaluated on the basis of TS and RS contents using Eq. 1.

(1) $$\begin{array}{l} \text{SD}=\left\{\text{DS/TS}\right\}\times 100\%\text{ or }100\%-\left\{\left(\text{Resistant starch/Total starch})\times 100\%\right)\right\} \end{array}$$

where SD = *in vitro* digestibility of starch (percentage of TS hydrolyzed) (19).

### Preparation of Plant Extracts for α-Amylase Inhibition, Total Phenol, and Flavonoid Assays

About 10 g of the raw FS were weighed into different flasks. Subsequently, the samples were boiled in 50 mL of distilled water following the procedures as described for sample preparation. For the unprocessed (without boiling or peeling or both as applicable), 10 g were prepared in 50 mL of distilled water. After the samples had been cooked, they were made up to 50 mL with distilled water, cooled under room temperature, and then homogenized in the liquids (water) they were dissolved in. The raw samples were homogenized in a similar way as the boiled samples. The homogenized samples were filtered through Whatman filter paper and evaporated (concentrated) at 50°C using a steam bath until most of the filtrate had been evaporated. Some quantities of the extracts were used for total phenol and flavonoid assays while the rest were reconstituted in distilled water to the concentrations: 10, 20, 30, 40, and 50 mg/mL, respectively for α-amylase inhibition assays.

### Assay of α-Amylase Inhibition

The α-amylase inhibitory actions of the aqueous extracts of the raw and cooked FS were determined as previously described (15, 20). Measured quantities (25 µL) of each diluted extract were mixed with 25 µL of 20 mM sodium phosphate buffer (pH 6.9) containing porcine α-amylase (0.5 mg/mL) (Cat. No. A3176, Sigma-Aldrich Chemical Co., Germany) in different test tubes and the reaction mixtures were kept at 25°C for 10 min. Thereafter, 25 µL of 0.5% starch in 20 mM sodium phosphate buffer was added to each test tube. The mixtures were kept at 25°C for 10 min after which the reaction was stopped by the addition of 50 µL of 96 mM dinitrosalicyclic acid color reagent. The reaction mixtures were then kept in a boiling water bath for 10 min, cooled at room temperature, and the absorbance was read at 540 nm using a UV spectrophotometer (UV-2100, UNICO). The α-amylase inhibitory capacities of the extracts were expressed as a percentage using Eq. 2.

(2) $$\begin{array}{l} \text{Percent inhibition }=\{(\text{Absorbance of control}\\ \;-\text{Absorbance of sample})\text{/Absorbance of control}\}\times 100. \end{array}$$

The absorbance of the control represents the absorbance without the extract to give 100% enzyme activity. By removing the sample (extract) from the solution, interferences arising from the extract such as color or self-inhibitory interferences were removed (21). Acarbose solution (at the concentrations 2, 4, 6, 8, 10 mg/mL) was used as a positive control. The blank was prepared by replacing the enzyme with the buffer solution (to allow for the absorbance produced by the plant extract) (22).

### Assay for Total Phenols

The total phenol contents of the raw and cooked FS were determined following the method of Singleton et al. (23). The extract (50 mg) was dissolved in 10 mL of water. To 0.1 mL of the sample solution was added 50 µL of Folin-ciocalteu’s reagent and the reaction mixture was well shaken for thorough mixing. After 3 min, 0.3 mL of 20% Na_2_CO_3_ (in water) was added to the setup and the whole mixture was shaken and incubated for 15 min at room temperature after which the absorbance was recorded at 725 nm. Gallic acid standard solution at the concentrations 2, 4, 6, 8, and 10 mg/mL, respectively was used to prepare a standard curve and the concentration of phenols in the samples was quantified from the curve. Results were converted and reported as g gallic acid equivalence/100 g fresh weight basis.

### Assay for Total Flavonoids

The total flavonoid contents of the raw and cooked FS were determined using the method of Ordon et al. (24, 25). The extract (100 mg) was dissolved in 10 mL of water. To 0.5 mL of the sample solution, 0.5 mL of 2% AlCl_3_ in ethanol was added and the mixtures were incubated for 1 h at room temperature. Thereafter, the absorbance of the samples was measured at 420 nm using a spectrophotometer. A standard solution of quercetin at the concentrations 10, 20, 30, 40, and 50 mg/mL was used to prepare a standard curve and the amount of flavonoids in the samples were determined from the standard curve. Results were converted and reported as g quercetin equivalence (QE)/100 g fresh weight basis.

### Statistical Analysis

Data were analyzed statistically using the Statistical Package for Social Sciences version 20.00. Results were presented as means ± SD of triplicate experiments. One-way analysis of variance was used for comparison of the means. *Post hoc* tests were carried out using the New Duncan Multiple Range Test. Differences between means were considered to be significant when *P* < 0.05.

## Results

The results for the TS, RS, DS, and SD of the raw and cooked FS are presented Table 1. Significant increases (*P* < 0.05) in the TS contents of boiled *R. virescens, A. auricula-judae*, unpeeled, and peeled potato, respectively were observed when compared with their raw. On the other hand, the TS contents of boiled sweet potato decreased significantly (*P* < 0.05) when compared with the raw.

**Table 1:** Total, resistant, digestible starch (g/100 g), and percentage starch digestibility of raw and boiled mushrooms, potato, and sweet potato.

Groups	TS	RS	DS	% starch digestibility
Raw *Russula virescens*	21.70 ± 0.14^d^	0^a^	21.70 ± 0.14^f^	100.00 ± 0.01^g^
Boiled *R. virescens*	64.90 ± 0.99^f^	2.20 ± 0.85^b^	62.70 ± 1.84^g^	96.60 ± 1.36^f^
Raw *Auricularia auricula-judae*	18.80 ± 1.41^b^	0^a^	18.80 ± 1.41^d^	100.00 ± 0.00^g^
Boiled *A. auricula-judae*	21.5 ± 0.14^d^	3.5 ± 0.99^c^	18.00 ± 0.85^d^	83.74 ± 4.50^d^
Raw unpeeled potato	18.70 ± 0.71^b^	10.10 ± 0.42^f^	8.60 ± 0.28^b^	45.99 ± 0.23^a^
Boiled unpeeled potato	24.30 ± 0.14^e^	4.50 ± 0.14^d^	19.80 ± 0.28^e^	81.49 ± 0.47^d^
Raw peeled potato	18.70 ± 0.14^b^	5.60 ± 0.57^d^	13.10 ± 0.42^c^	70.07 ± 2.79^c^
Boiled peeled potato	20.10 ± 0.14^c^	7.10 ± 0.14^e^	13.00 ± 0.28^c^	64.68 ± 0.45^b^
Raw sweet potato	21.90 ± 0.14^d^	2.20 ± 0.00^b^	19.70 ± 0.14^e^	89.96 ± 0.06^e^
Boiled sweet potato	6.40 ± 0.00^a^	0^a^	6.40 ± 0.00^a^	100.00 ± 0.00^g^

*Values are means ± SD*.

*^a–g^Means with different superscript letters along each column are significantly different (*P* < 0.05)*.

*TS, total starch; RS, resistant starch; DS, digestible starch; rapidly digestible starch*.

As shown in Table 1, the RS contents of the raw and cooked FS ranged from 0 to 10.10/100 g. Whereas RS was not detected in boiled sweet potato and the raw forms of *R. virescens* and *A. auricula*-judae, boiling of *R. virescens, A. auricula-judae*, and peeled potato resulted in significant increase (*P* < 0.05) of RS compared with their raw.

The DS contents of the raw and boiled FS ranged from 6.4 to 62.7 g/100 g. There were significant increases (*P* < 0.05) in the DS contents of boiled *R. virescens* and boiled unpeeled potato compared with the raw; no significant differences (*P* > 0.05) in the DS contents of boiled *A. auricula-judae* and boiled peeled potatoes compared with the raw; but significant decreases (*P* < 0.05) in the DS contents of the boiled sweet potato samples compared with their raw.

As shown in Table 1, the percentage of SD of the raw and cooked FS ranged from 45.99 to 100% while raw *R. virescens*, raw *A. auricula-judae*, and boiled sweet potato had the highest percentage of SD, raw unpeeled potato had the least.

The results of the *in vitro* α-amylase inhibitory actions of the aqueous extracts of the raw and cooked FS are shown in Table 2.

**Table 2:** α-Amylase inhibitory properties (%) of aqueous extracts of raw and boiled mushrooms, potato, and sweet potato.

Groups	Percentage α-amylase inhibition
Raw *Russula virescens*	−121.22 ± 15.81^b^
Boiled *R. virescens*	0.00^d^
Raw *Auricularia auricula-judae*	19.75 ± 4.96^f^
Boiled *A. auricula-judae*	−1.95 ± 0.23^c^
Raw unpeeled potato	39.02 ± 1.81^g^
Boiled unpeeled potato	62.20 ± 23.08^i^
Raw peeled potato	18.54 ± 4.44^e^
Boiled peeled potato	50.24 ± 6.07^h^
Raw sweet potato	64.14 ± 4.22^j^
Boiled sweet potato	−131.39 ± 56.37^a^
Acarbose (2–10 mg/mL)	69.76 ± 21.40^k^

*Values are means ± SD*.

*^a–k^Means with different superscript letters along each column are significantly different (*P* < 0.05). Concentration of the food samples for α-amylase inhibition assay 10–50 mg/mL*.

As shown in the Table, extracts of raw *A. auricula-judae*, unpeeled potato, peeled potato, sweet potato, and boiled unpeeled and peeled potato showed mild to high inhibitory properties on α-amylase and which inhibitory properties (expressed as a percentage) ranged from 18.54 to 64.14% (over a concentration range of 10–50 mg/mL) as compared to that of the standard antidiabetic drug (acarbose) that had 69.08% inhibition of α-amylase activity over a range of concentration of 2–10 mg/mL. Furthermore, extracts of raw *R. virescens*, boiled *A. auricula-judae*, and sweet potatoes had negative to 0 values.

There was a significant negative correlation between the RS contents of the raw and cooked FS versus their SD (*r* = −0.97) (Table 3).

**Table 3:** Correlations between SD versus RS and α-amylase inhibition; RS versus α-amylase inhibition; α-amylase inhibition versus total phenol and flavonoid of the food samples.

	RS	α-amylase inhibition	Phenol	Flavonoid
SD	−0.97**	−0.55*	ND	ND
RS	ND	0.59**	ND	ND
α-amylase inhibition	ND	ND	0.11	0.39

**Correlation is significant at P = 0.01 level.

*Correlation is significant at P = 0.05 level.

*RS, resistant starch; SD, starch digestibility; ND, not determined*.

**Figure 1:**
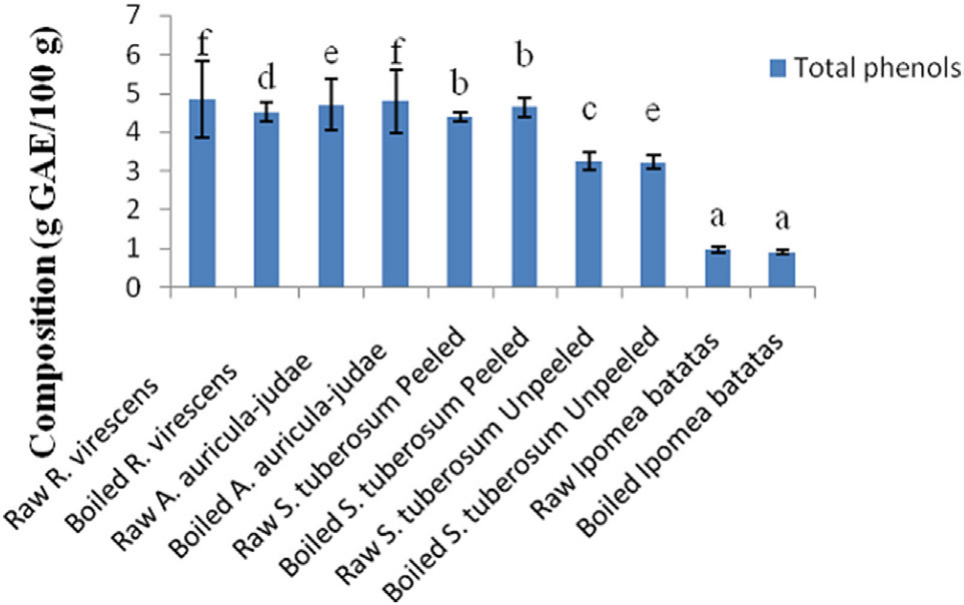
Total phenol contents of aqueous extracts of raw and boiled mushrooms, potato, and sweet potato. Values are reported as means ± SD. (a–f) Means with different superscripts are significantly different (*P* < 0.05) across the groups. GAE, gallic acid equivalence.

**Figure 2:**
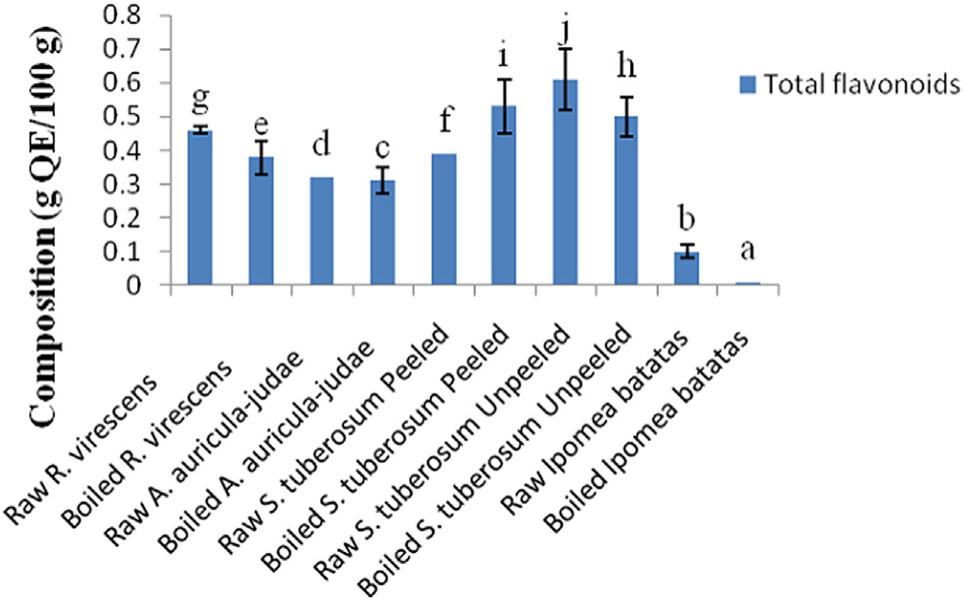
Total flavonoid contents of aqueous extracts of raw and boiled mushrooms, potato, and sweet potato. Values are reported as means ± SD. (a–j) Means with different superscripts are significantly different (*P* < 0.05) across the groups. QE, quercetin equivalence.

The total phenol and flavonoid contents of aqueous extracts of the raw and cooked FS are shown in Figures 1 and 2. The total phenol contents (g gallic acid equivalents/100 g) of the raw and cooked FS were: 4.85 ± 0.98 for raw *R. virescens*, 4.52 ± 0.24 for boiled *R. virescens*, 4.71 ± 0.66 for raw *A. auricula-judae*, 4.80 ± 0.81 for boiled *A. auricula-judae*, 4.40 ± 0.11 for raw *S. tuberosum* peeled, 4.64 ± 0.26 for boiled *S. tuberosum* peeled, 3.26 ± 0.22 for raw *S. tuberosum* unpeeled, 3.23 ± 0.18 for boiled *S. tuberosum* unpeeled, 0.99 ± 0.08 for raw *I. batatas*, and 0.92 ± 0.05 for boiled *I. batatas*. The total flavonoid contents (g QE/100 g) of the raw and cooked FS were: 0.46 ± 0.01 for raw *R. virescens*, 0.38 ± 0.05 for boiled *R. virescens*, 0.32 ± 0.00 for raw *A. auricula-judae*, 0.31 ± 0.04 for boiled *A. auricula-judae*, 0.39 ± 0.00 for raw *S. tuberosum* peeled, 0.53 ± 0.08 for boiled *S. tuberosum* peeled, 0.61 ± 0.09 for raw *S. tuberosum* unpeeled, 0.50 ± 0.06 for boiled *S. tuberosum* unpeeled, 0.10 ± 0.01 for raw *I. batatas*, and 0.01 ± 0.00 for boiled *I. batatas*.

There were significant decreases (*P* < 0.05) in the total phenol and flavonoid contents of boiled *R. virescens* compared with the raw.

There were significant increases (*P* < 0.05) in the total phenol but significant decreases (*P* < 0.05) in the total flavonoid contents of boiled *A. auricula-judae* and unpeeled potato compared with their raw. There were non-significant differences (*P* > 0.05) in the total phenol contents of boiled peeled potato when compared with the raw (Figure 1) but significant increases (*P* < 0.05) in the total flavonoid contents of boiled peeled potato when compared with the raw (Figure 2).

As shown in Figure 2, there were significant decreases (*P* < 0.05) in the total flavonoid contents of boiled sweet potato when compared with the raw. There was a non significant positive correlation between the alpha amylase inhibitory properties of the aqueous extracts of the raw and cooked FS versus their total phenol (*r* = 0.11) and flavonoid (*r* = 0.39) contents (Table 3).

As shown in Table 3, there was a significant negative correlation (*r* = −0.55; *P* = 0.05) between the α-amylase inhibitory properties of the raw and cooked FS versus their SD and a significant positive correlation (*r* = 0.59; *P* = 0.01) between the α-amylase inhibitory properties of the raw and cooked FS versus their RS contents.

## Discussion

The methods of processing that were used in this study were selected as they represent the ways in which the FS that were investigated in this study are eaten in typical African diets.

The increases in the TS contents of boiled *R. virescens, A. auricula-judae*, unpeeled and peeled potato could be associated with the partial loss of soluble components in some cooked foods (25). Similar reports were given by Capriles et al. (26) and Małgorzata et al. (27) on cooked/boiled *Amaranthus cruentus* seeds, and beans respectively. However, the decreased TS contents of boiled sweet potato suggest that the starch granules in the sweet potato tubers may have been damaged during the cooking and homogenizing processes. When the effect of boiling and peeling of the potato tubers on their TS contents was considered, it was observed that raw unpeeled and peeled potatoes had higher TS contents than boiled unpeeled and peeled potatoes respectively.

Resistant starch refers to the fraction of dietary starch that escapes digestion in the small intestine (28). In addition to GI, RS has also been established as an important index of measurement of SD. Although boiling of starchy foods leads to gelatinization of starch, cooling causes a rearrangement of the starch molecules, making part of the starch to become resistant to digestion (15) due to the formation of RS type 3. Thus, it is possible that greater amounts of RS type 3 may have been formed in the boiled *R. virescens, A. auricula-judae*, and peeled potato samples during the period they were cooled. On the other hand, the loss of RS in boiled sweet potato suggests that the effect of retrogradation may have been slower in sweet potato and further affirms that the starch granules in the sweet potato tubers may have been damaged during the cooking and homogenizing processes. It was observed in this study that despite increased amount of TS in boiled unpeeled potatoes, they had lower amounts of RS compared with their raw. While the reason for this cannot be explained presently, it is noteworthy. When the effect of boiling and peeling of the potato tubers on their RS contents was compared, it was observed that raw unpeeled potato had higher amounts of RS than boiled unpeeled potato, raw and boiled peeled potatoes, respectively.

When the DS values of the FS were expressed as percentages of their TS hydrolyzed (reported as SD of the samples), it was observed that 45.99–100% of the TSs in the raw and boiled FS were hydrolyzed. The higher percentage of SD in raw *R. virescens, A. auricula-judae*, and boiled sweet potatoes compared with other FS investigated could be attributed to the non-detectable levels of RS in them. Furthermore, the higher percentage of SD in boiled unpeeled potato compared with the raw form could be attributed to its loss of RS during the boiling process as cooking of foods has been shown to affect the digestibility of starches in foods (4, 15). On the contrary, the lower percentages of SD in boiled *R. virescens, A. auricula-judae*, and peeled potato compared with their raw could be attributed to the increased amount of RS in them when they were boiled. When the effect of boiling and peeling of the potato tubers on the digestibility of their starches was considered, it was observed that raw unpeeled potato had lower percentage of SD than boiled unpeeled potato, raw and boiled peeled potatoes due to higher amounts of RS. Since sweet potatoes are also eaten in the raw form in Nigeria, the findings of this study reveals that consumption of the raw form of unpeeled potato will be of more benefit to people with type 2 diabetes than consumption of the boiled unpeeled form, raw and boiled peeled forms, respectively.

In addition, the significant negative correlation obtained between the RS versus SD of the raw and processed mushroom, potato, and sweet potato samples further affirms that the RS contents of these FS significantly influenced their SD.

Inhibition of pancreatic α-amylase is one of the therapeutic targets for delaying oligosaccharide digestion to absorbable monosaccharides in the intestine, resulting in reduced postprandial hyperglycemia (1). Therefore, inhibition of α-amylase by aqueous extracts of raw *A. auricula-judae*, raw unpeeled potato, boiled unpeeled potato, raw peeled potato, boiled peeled potato, and raw sweet potato suggests the potential beneficial use of these plants in the management of diabetes. Furthermore, the zero inhibition demonstrated by boiled *R. virescens* suggests the inability of the boiled form of this mushroom variety to inhibit α-amylase under *in vitro* conditions. On the contrary, the negative inhibition shown by extracts of *R. virescens*, boiled *A. auricula-judae*, and boiled sweet potatoes indicate that the α-amylase was activated rather than being inhibited by the tested extracts of these FS. If this were to occur *in vivo*, it would aggravate, rather than ameliorate the diabetic condition as it could cause a spike in blood glucose (22).

Phenolics and flavonoids belong to the class of polyphenols that have received considerable attention because of their antioxidant potentials and effects on carbohydrate metabolism involving the inhibition of α-amylase and α-glucosidase (17). The significant decreases in the total phenol and flavonoid contents of boiled *R. virescens* compared with the raw suggest that they may either have leached into the boiling vessel or may have been denatured as a result of heat treatment.

The increased amount of phenols following boiling of *A. auricula-judae* and unpeeled potato suggests the release of some bound phenols from their food matrix. Similar observations were made in *Solanum paniculatum* fruit (29). On the other hand, the decreased amount of flavonoids in boiled *A. auricula-judae* and unpeeled potato suggest that they may either have been denatured or leaked into the boiling vessel.

The non-significant differences in the total phenol contents of boiled peeled potato when compared with the raw suggest that the cooking method that was used for the peeled potato samples preserved their phenolic compounds. Furthermore, the increases in the total flavonoid contents of boiled peeled potato when compared with the raw suggest better extraction of these compounds in the boiled peeled potato than the raw. In addition, the non significant differences in the total phenol contents of boiled sweet potato when compared with the raw suggest that the cooking method preserved the phenol constituents of sweet potato.

We assume the decreases in the total flavonoid contents of boiled sweet potato could be due to their breakdown or these polar flavonoids could have leaked into the boiling water. When the effect of boiling and peeling of the potato tubers on their polyphenol contents was considered, it was observed that boiled unpeeled potato had higher amounts of total phenols than raw unpeeled, raw, and boiled peeled potatoes, respectively, suggesting that boiling of unpeeled potato enhanced better extraction of its bound phenols than the raw unpeeled, raw, and boiled peeled potatoes, respectively. On the other hand, raw unpeeled potato had higher amounts of flavonoids than boiled unpeeled, raw, and boiled peeled potatoes, respectively.

The non significant positive correlation between the percentages of inhibition of the aqueous extracts of the raw and cooked FS versus their total phenol and flavonoid content suggest that the inhibitory actions of the tested extracts of some of these FS on α-amylase may be mediated by the action of constituents other than their phenol and flavonoid constituents, such as dietary fiber and others. Similar reports were also given by Oyedemi et al. (1).

It has been suggested that there could be a relationship between the α-amylase inhibitory actions of some plant based foods versus their SD (30). Therefore, the significant negative correlation between the α-amylase inhibitory properties of the raw and cooked FS versus their SD lends credence to this as it indicates that the α-amylase inhibitors in these FS also influenced the digestibility of their starches. Furthermore, the significant positive correlation between the α-amylase inhibitory properties of the raw and cooked FS versus their RS contents indicates that the RS constituents of these FS are important contributors to their α-amylase inhibitory properties.

To the best of our knowledge, this is the first report on the effect of cooking on SD, polyphenol contents, and *in vitro* α-amylase inhibitory properties of mushroom; the effect of cooking of sweet potato on the digestibility of its starch and the activities of starch blockers that could be present in it; and the effect of cooking on SD, polyphenol contents, and *in vitro* α-amylase inhibitory properties of potato, which makes this study novel.

## Conclusion

The raw form of *Russula virescens* and boiled sweet potato had non-detectable levels of RS in them, which made their starches to be highly digestible. Furthermore, they activated pancreatic α-amylase rather than inhibiting it under *in vitro* condition, suggesting that they may not be useful as functional foods for people with type 2 diabetes mellitus. However, boiled unpeeled potato, boiled peeled potato, and raw sweet potato demonstrated considerable degrees of α-amylase inhibitory properties under *in vitro* conditions suggesting their usefulness as functional foods for people with type 2 diabetes.

## Recommendations for Future Studies

It is recommended that the results of these studies be confirmed using *in vivo* studies.

## Author Contributions

CE conceived/designed the work. CE, SS, DE, AI, MB, and NC contributed to the acquisition, analysis, interpretation of data for the work; contributed to the drafting of the work and critically revised it for important intellectual content. All authors gave their final approval of the version to be published.

## Conflict of Interest Statement

The authors declare that the research was conducted in the absence of any commercial or financial relationships.
